# Editorial: Transplantation of Marginal Organs—Immunological Aspects and Therapeutic Perspectives

**DOI:** 10.3389/fimmu.2020.612576

**Published:** 2020-10-29

**Authors:** Caner Süsal, Thomas Friedrich Mueller, Christophe Legendre, Peter Schemmer

**Affiliations:** ^1^ Institute of Immunology, Heidelberg University Hospital, Heidelberg, Germany; ^2^ Clinic for Nephrology, University Hospital Zürich, Zürich, Switzerland; ^3^ Service de Transplantation Rénale et Unité de Soins Intensifs, Hôpital Necker-Enfants Malades, AP-HP, Paris Descartes University, Paris, France; ^4^ General, Visceral and Transplant Surgery, Department of Surgery, Medical University of Graz, Graz, Austria

**Keywords:** organ transplantation, marginal donors, expanded criteria donors, machine perfusion, organ allocation, graft survival, delayed graft function, antigen silencing

In organ transplantation, shortage of available grafts has resulted in a continuously growing use of kidneys, livers, lungs, and hearts from elderly donors, often with comorbidities. The percentage of ≥60-year-old deceased kidney donors reported to the Collaborative Transplant Study (CTS) from Europe was 21% during 2000 to 2001 and increased to as high as 42% during 2016 to 2017 (1). Due to a decreased number of functional tissue and inflammation-mediated alloantigen expression, these marginal organs from expanded criteria donors (ECD) are considered to be more vulnerable to immune attack and show impaired survival rates. Different criteria are used in different organ types to define ECDs. In kidney transplantation, besides a donor age above 59, a history of hypertension, increased creatinine and cerebrovascular cause of death are key factors impacting the quality of the deceased donor organ. Articles in this Research Topic highlight the problems that are encountered in transplantation of marginal organs and propose novel diagnostic and methodological approaches, including improvement of organ allocation strategies, use of alternative therapeutic regimens and utilization of modern machine perfusion (MP) techniques that enable estimation of organ quality, preconditioning of donors, depletion of immune cells and genetic silencing of alloantigens.

Gerbase-DeLima et al. underline with their large single-center analysis of more than 5,000 kidney transplantations the importance of the awareness of risk that is associated with different combinations of donor and recipient age. They report lower death censored graft and patient survival in recipients of kidneys from elderly donors and higher graft but lower patient survival in elderly recipients. Also factors, such as time on dialysis and HLA match, influenced outcome differentially in different recipient and donor age combinations. Noble et al. describe in their comprehensive review immunological aspects of kidney transplantation in elderly recipients with organs from marginal donors and highlight therapeutic options that could help to prevent side effects, such as toxicity and development of cancer. Echterdiek et al. report, despite a negative trend in demographic parameters, an improving outcome of kidneys from ≥70-year-old donors in Europe over the years, most probably due to an improving physiology of elderly donors. This finding could encourage to increase the donor pool also in other parts of the world, such as the United States, where organs from elderly donors are currently discarded at a high rate.

Eurotransplant (ET) Senior Program (ESP) is an “old-for-old” allocation scheme in which kidneys from ≥65-year-old deceased donors are allocated independent of HLA matching to ≥65-year-old recipients locally in order to prevent further ischemic injury in an already vulnerable organ during the transport to a better matched recipient. Increasingly more kidney transplantations are currently performed *via* ESP and their percentage among all performed deceased donor kidney transplantations rose in ET countries from 9% in 2000 to 18% in 2019 (2). Dreyer and de Fijter advocate in their review the introduction of a restricted form of matching in ESP for only the HLA-DR locus instead of the three HLA loci that are considered in regular kidney allocation. They claim that this procedure can be performed locally without prolonging the cold ischemia time (CIT) and is expected to result in less rejections and mortality. Prolonged CIT is also in liver transplantation a critical factor that affects graft and patient survival. Lozanovski et al. found in their CTS analysis of more than 40,000 liver transplant recipients that the negative influence of CIT on outcome is much stronger in patients with hepatitis C-related cirrhosis than for example in patients with alcoholic cirrhosis or in patients with hepatocellular carcinoma and low “model of end-stage liver disease” scores. They, therefore, suggest that original disease should also be implemented as a criterion in allocation of livers.

Due to the general trend of increasing donor age, delayed graft function (DGF) is observed in a growing proportion of recipients of deceased donor kidney transplants. Morath et al. analyzed the influence of DGF in association with pre-sensitization in kidney transplantations performed between 2008 and 2017. Besides the known non-immunological factors, such as high donor age and prolonged ischemia time, the pre-transplant presence of broad alloantibody reactivity was found to be a significant predictor of DGF, also in this more recent era of transplantation during which sensitive antibody testing was practiced. Development of DGF itself doubled the risk of graft loss which, however, increased further if the patient had HLA or donor-specific HLA antibodies before transplantation. These findings indicate that special measures are necessary during allocation of organs from elderly donors, especially to pre-sensitized patients.

Robust measures of organ quality are required for reliable prediction of successful outcomes in the use of marginal organs. However, organ quality is difficult to assess, in particular, within the narrow window of time to transplantation. In this setting molecular diagnostics could complement the clinical and histopathological evaluation of tissue quality by capturing additional information on immune- and non–immune-mediated injury as well as repair mechanisms. Von Moos et al. reviewed the current state of quality assessment in donor kidneys using clinical scores, histopathology and perfusion characteristics with an emphasis on molecular analyses. They describe shortcomings of available methods and studies. The review highlights advances made in the integration of molecular analyses with clinical data and future studies necessary to perform. Along the same line, Hruba et al. investigated in different donor categories transcriptome profiles of allograft biopsies with borderline changes. When compared with standard criteria or living donor kidneys, ECD kidneys showed higher expression of transcripts related to inflammation and extracellular matrix remodeling. These changes are expected to aggravate alloimmune responses and influence outcomes.  Boissier et al. compared the cellular components and the transcriptomic and vasculogenic profiles in the peri-renal adipose tissue of kidneys from ECDs and optimal donors. Peri-renal adipose tissue of ECDs was found to display an age-dependent inflammatory signature that was associated with early graft dysfunction. NK-cell subsets were recruited differentially in the peri-organ environment of kidney grafts from elderly donors. This novel evidence indicate that peri-renal adipose tissue, which can be gained non-invasively and timely, represents a valuable source of donor material to assess inflammatory changes that affect organ quality and function. Corradetti et al. highlight in their case report cholesterol embolism as a rare but severe adverse event that can occur in transplantation of marginal donor kidneys and report its successful treatment with the prostaglandin I2 analog iloprost. Causality still has to be shown here and controlled studies are necessary to assess the true value of the iloprost therapy.

MP is on the way of becoming a core technology in the use of ECD organs that allows not only the estimation of organ quality prior to transplantation but also enables intervention for improved preservation, (re)conditioning and regeneration of organs. Furthermore, it has the potential for a medical revolution toward organ engineering. Resch et al. review in detail the beneficial use of oxygenated hypothermic and normothermic MP and describe findings which indicate that MP prolongs the graft preservation time significantly and allows, during the perfusion procedure, treatments generating chimeric organs and enabling immunological changes, defatting, reduction of inflammation and elimination of hepatitis C. They state that improved graft function after MP can most likely be explained by amelioration of ischemia/reperfusion injury (IRI) and assessment of the graft’s viability and function prior to transplantation. Kvietkauskas et al. summarize in their review experimental studies and clinical trials on MP-associated treatment strategies and focus hereby on inhibition of allorecognition pathways. Specifically, they show evidence that MP protects the organ from inadequate activation of innate immunity by decreasing IRI. Unfortunately, established clinical standards in MP-protocols are still missing.

In several experimental models, MHC-silenced cells were shown to be protected against allogeneic immune responses (3, 4). Yuzefovych et al. demonstrate a sub-normothermic ex vivo perfusion system in rats which enables the delivery of lentiviral vectors that encode small hairpin RNAs to permanently silence MHC antigens. If feasible and safe in humans, generation of immunologically silenced organs raise great expectations to solve the major problems of organ transplantation, such as rejection and side effects of immunosuppressive regimens. Donor hearts have significant leukocyte reservoirs which can activate recipient leukocytes and initiate acute rejection upon transplantation. Critchley et al. demonstrate in an experimental porcine heart transplantation model that, as compared to static cold storage, hypothermic MP with cardioplegic solution reduces immunogenicity of the organ significantly *via* elimination of resident leukocytes. Besides a pro-inflammatory cytokine pattern, a pro-survival- and reduced ischemia-related profile was observed after hypothermic MP that could explain the improvement in graft viability.

In addition to MP-technology, the clinical expertise of transplant surgeons is of great importance to both accept and allocate organs from ECDs to suitable recipients. Kahn et al. compared the outcome of liver transplants from ECDs versus non-ECDs and show evidence that, if a well-standardized allocation strategy based on clinical facts is used, the outcome of ECD livers is not compromised with a 90-day mortality of only 3.6%.

Dezfouli et al. compared, in a large animal model, anti-inflammatory effects of preconditioning of kidney donors after brain death with different therapeutic regimens. Interestingly, oral administration of calcineurin inhibitors as well as inhibitors of mammalian target for rapamycin decreased TNF-alpha expression more effectively than the routinely administered intravenous steroids, indicating that it would be worth to investigate in additional studies the protective effect of oral donor preconditioning on IRI.

The present Research Topic proves that transplantation of ECD organs has become a major issue in organ transplantation and describes solutions to the problem.

## Author Contributions

CS, TFM, CL, and PS wrote the manuscript. All authors contributed to the article and approved the submitted version.

## Conflict of Interest

The authors declare that the research was conducted in the absence of any commercial or financial relationships that could be construed as a potential conflict of interest.
